# Mucous Polypi of the Maxillary Sinus

**Published:** 1859-01

**Authors:** 


### ARTICLE IV.

*Mucous Polypi of the Maxillary Sinus.* Translated for the American Journal of Dental Science.

PROFESSOR LUSCHKA has ascertained, after extensive research, that the assertion made by M. Billroth in his work entitled "De la Structure des Polypes Muqueux," is far from being well founded; viz. that the mucous membrane of the maxillary sinus is only very rarely the seat of soft polypus. Take a thread of the membrane covering the antrum of Highmore, which has been detached by the preparation, or which has been preserved in alcohol, and you will easily discover that this membrane is composed, properly speaking, of the mucous and periosteal tissues. The mucous tissue is very fine, delicate and transparent, easily torn, and very glossy on its free surface. It is very loosely united by cellular tissue to the periosteum, from which it may be readily detached; generally but few glands are found. The periosteum, on the contrary, has much more resistance, being nearly double the thickness; it is also opaque when fresh. It can be detached from the bones without difficulty, ordinarily, yet it is by no means rare to find it adhering in places with the bony tissues. Little stalactiform processes enter into the meshes of its tissue, and give it a slightly mammilated appearance. The dental nerves inclosed in the canaliculi or grooves, are immediately under the periosteum. It is not rare to meet with odontologiæ more or less violent, succeeding a nasal catarrh which has arisen from the membrane of the maxillary sinus, although the teeth are perfectly sound; in such cases the inflammation causes a compression of the nerves, which is followed by the swelling of the membrane.*

The glands are the most remarkable constituents of this

* Catarrhal Odontologiæ of the ancients.

fibrous tissue, the existence of which have been completely denied, or which have only been admitted to exist in very small numbers. These glands, which have the form of branching tubes, and are about two millimetres long, appear in adults with swellings like blisters, which may degenerate and form large cysts, which we so often find in the maxillary sinus. Smaller cysts are also found, some the size of hemp seed, others as large as a hazel nut. Sometimes alone, sometimes in great numbers, these vesicles are sessile or provided with a pedicle at the expense of the membrane which they have involved; they advance also more or less towards the centre of the cavity. The smaller ones generally contain a seedy mass of the consistence of cooked sago, with very fine rounded fatty aggregated granulations. As to the larger vesicles, they contain a clear yellow liquid, with cheesy lump, lumps in which besides the above qualities are found crystals of fat and starchy matter. Their parietes present a very diverse structure; sometimes they are formed by a homogeneous pellicle transparent as glass, at other times by the elements of cellular tissue at different degrees of development, with an incomplete epithelium having undergone fatty degeneration. It is not so rare, though not frequently, that we find polypus covered by the mucous membrane and supported by the growth of the submucous cellular tissue. Sessile and pediculated, they are supported by the loose cellular tissue, or are contained in a gelatine-form substance. They are generally of a knotty or periform shape with thin prolongations, or attenuated like a fibre; rarely, at least to the naked eye, can depressions or cavities be perceived. Their length varies from one and a half to two centimetres. Most frequently situated on the internal parietes of the sinus, they intercept communication with the cavity of the lesser cornet, and cause accumulation of mucus. But on account of their extreme smallness, these polypi must be in great number to produce this effect.

Dr. G. . . . [*L'Art Dentaire.*

